# Point of Care Ultrasound Diagnosis of Maxillary Artery Pseudoaneurysm in the Emergency Department

**DOI:** 10.24908/pocus.v9i1.16831

**Published:** 2024-04-22

**Authors:** Marwa L Ali, Sean Beckman, Stephen Alerhand

**Affiliations:** 1 Department of Emergency Medicine, Rutgers New Jersey Medical School Newark, NJ USA

**Keywords:** Maxillary artery pseudoaneurysm, pseudoaneurysm, point-of-care ultrasound, POCUS

## Abstract

A pseudoaneurysm results from a tear in a vessel wall. This leads to extravasation of blood into adjacent tissue and eventual formation of a fibrous sac that maintains continuity with the lumen. These vascular injuries very rarely occur in deeper vessels of the face (e.g. maxillary artery) due to protection from structures like the bony mandible and parotid gland. If left untreated, these pseudoaneurysms can lead to infection, thromboembolism, hemorrhage, and compression of surrounding structures such as facial nerve branches. Pseudoaneurysms are typically diagnosed by advanced imaging techniques including computed tomography angiography and magnetic resonance angiography. However, these tests require time to perform and interpret, are costly, and take place outside the patient care area. Computed tomography also confers ionizing radiation. Fortunately, point of care ultrasound (POCUS) is a readily available, dynamic imaging tool that can be performed at the bedside. Here we present the first known case report of a maxillary artery pseudoaneurysm diagnosed by POCUS in the emergency department. Early differentiation from a typical hematoma led to rapid management in the form of a compression bandage, as well as expedited consultation to the appropriate services.

## Introduction

An aneurysm refers to the dilation of a blood vessel (usually an artery) that involves all three layers of that vessel (i.e. the tunica intima, tunica media, and tunica adventitia). In contrast, a pseudoaneurysm forms from a complete tear through all three layers. This leads to extravasation of blood that is subsequently contained by the surrounding fibrous tissue 1, 2, 3, 4, 5. Generally, pseudoaneurysms present clinically as painful, pulsatile masses, oftentimes with neurologic deficits secondary to nerve compression 3, 6, 7. They can be diagnosed using ultrasound, computed tomography angiography (CTA), and magnetic resonance angiography (MRA) 1, 7, 8. Management of pseudoaneurysms includes both noninvasive (e.g. observation, ultrasound-guided compression) and invasive measures (e.g. endovascular embolization, open surgical ligation) 1, 3, 9. If left untreated, pseudoaneurysms can lead to complications such as infection, thromboembolism, hemorrhage, and compression of surrounding structures 5, 7.

Pseudoaneurysms of the maxillary artery are rare due to its anatomic protection by the bony mandible and parotid gland 3, 4, 10, 11, 12. Prior case reports have described maxillary artery pseudoaneurysms as a complication of maxillofacial fractures and surgeries, blunt and penetrating trauma, infection, and radiation therapy 1, 3, 10, 11, 13, 14, 15, 16, 17. There have also been cases reported from penetrating injury 4, 11, 14, 18, 19, 20, 21, 22. In all these instances, the diagnoses were made using CTA or MRA, both of which confer a delayed diagnosis, transfer of the patient outside the care area, and higher cost. CTA also confers ionizing radiation. There are no known cases of a maxillary artery pseudoaneurysm diagnosed immediately by point of care ultrasound (POCUS) at the bedside.

Here, we present the first known case of a maxillary artery pseudoaneurysm diagnosed by POCUS in the emergency department (ED), stemming from a penetrating stab wound to the face. Rapid bedside use of POCUS facilitated immediate diagnosis, differentiation from a typical hematoma, the application of a tight compression bandage, and expedited consultation to the appropriate services for potential definitive management. 

## Case Presentation 

**Figure 1:**
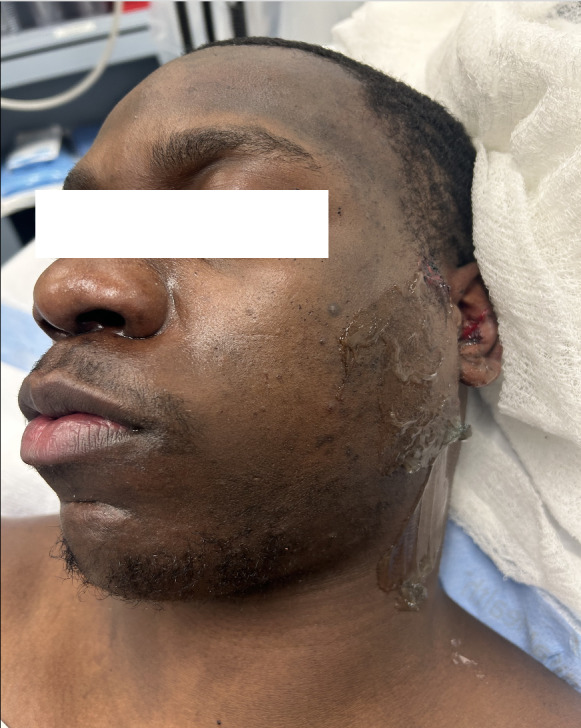
The patient had a large area of swelling to the left side of the face anterior to the tragus of the ear, along with a 2 cm laceration to the left cheek.

A 22 year-old man was brought in by ambulance to the ED after sustaining a stab wound to the left side of the face. Vital signs consisted of blood pressure 155/108 mmHg, heart rate 62 beats per minute, respiratory rate 12 breaths per minute, and 100% oxygen saturation on room air. On physical examination, the patient was in severe pain from the wound. There was a 2 cm laceration to the left cheek, along with a large area of swelling to the left side of the face anterior to the tragus of the ear (Figure 1).

**Figure 2:**
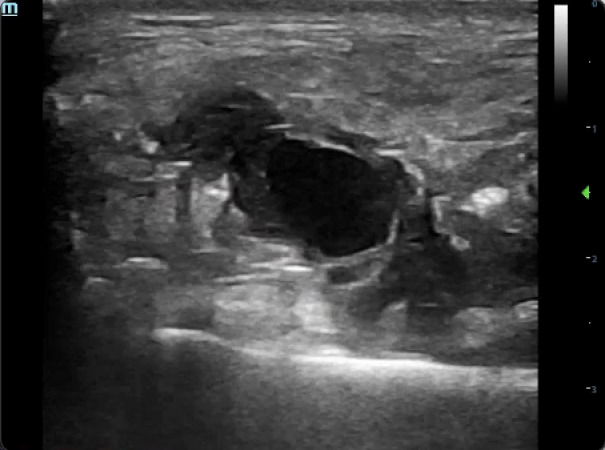
An anechoic, pulsatile, rounded, 2x2 cm structure with adjacent irregular pockets of internal echoes.

 Initial diagnoses that were considered included traumatic hematoma or parotid gland injury. Using the high-frequency (4-12 MHz) linear transducer, B-mode scanning revealed an anechoic, pulsatile, 2x2 cm rounded structure with adjacent irregular pockets of internal echoes (Figure 2) (Video S1).

**Figure 3:**
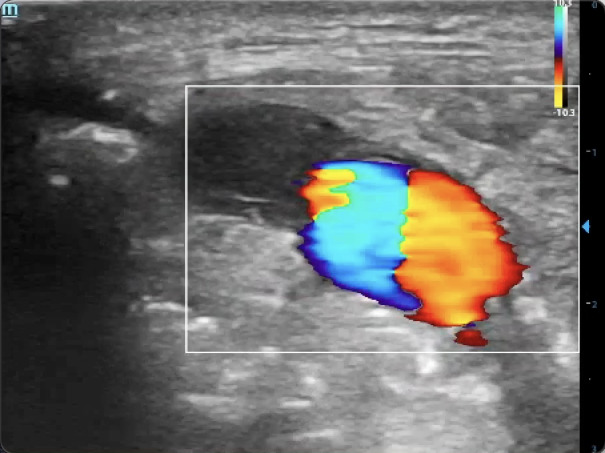
The “ying-yang” sign – a red and blue swirling pattern that appears from pulsatile blood being ejected from the arterial wall defect into the pseudoaneurysm sac, and then redirected back towards the neck by the surrounding fibrous tissue. The gain is properly set high enough to detect the change in Doppler shift from blood flow, but not too high that would result in appearance of artifact.

Color Doppler revealed the “ying-yang” sign – a red and blue swirling pattern resulting from pulsatile blood being ejected from the arterial wall defect into the pseudoaneurysm sac, and then redirected back S

**Figure 4:**
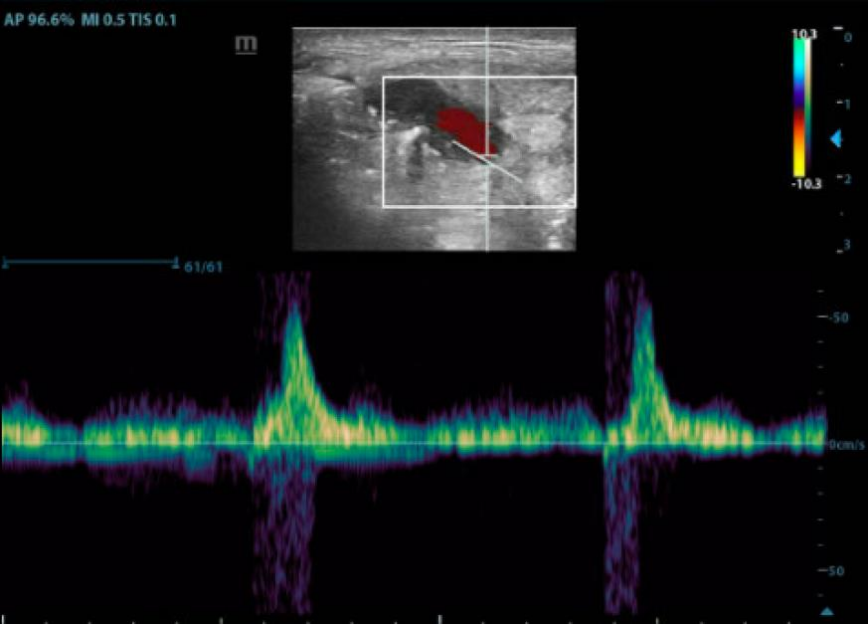
Pulsatile flow with the largest amplitude at the pseudoaneurysm neck. Not pictured is the decrease in amplitude towards the distal end of the sac. The gain is properly set high enough to detect the change in Doppler shift from blood flow, but not too high that would result in appearance of artifact. Also note how the scale along the right side of the screen is adjusted so that the waveform takes up as much of the screen as possible.

Pulsed wave Doppler revealed pulsatile flow with the largest amplitude at the pseudoaneurysm neck, with a decrease in amplitude towards the distal end of the sac (Figure 4).

Interestingly, upon holding compression and then releasing pressure upon the external carotid artery at the neck, the pseudoaneurysm could be visualized collapsing and re-expanding, respectively (Video S3). Altogether, these sonographic findings suggested the presence of a pseudoaneurysm. 

Especially given the patient’s worsening pain, POCUS findings prompted immediate placement of a tight compression bandage over the swelling. The Vascular Surgery service was consulted emergently, followed by a team discussion with the Oral and Maxillofacial Surgery and Neuroendovascular services as well. These teams requested a CTA, which confirmed the emergency physicians’ suspicion of the culprit maxillary artery based on the wound’s anatomic location. Given the deep location of the maxillary artery, the consultants decided to manage conservatively with a follow-up CTA in three days to evaluate for potential pseudoaneurysm expansion. At the subsequent 1-month follow-up visit, swelling of the face was no longer present on physical examination, and the patient denied symptoms.

## Discussion

This is the first known case report in which POCUS was used to immediately diagnose a maxillary artery pseudoaneurysm. The prompt diagnosis allowed for differentiation from the typical (and otherwise expected) hematoma, expedited management in the form of a tight compression bandage, early involvement of appropriate consulting services, and further diagnostic investigation of a potentially dangerous injury. Without immediate compression, there could have been continued pseudoaneurysm expansion, worsening pain from stretching of skin fibers, facial nerve compression and paralysis, and potentially hemorrhage.

**Figure 5:**
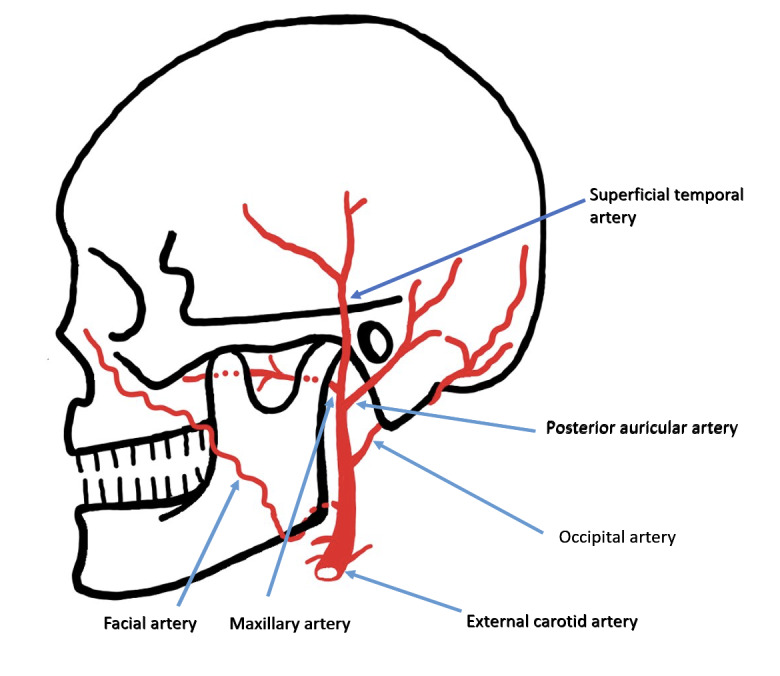
Pictorial diagram of the face’s arterial supply, including the maxillary artery.

The maxillary artery’s deep anatomic course renders it less susceptible to penetrating injury, so pseudoaneurysm formation from such injury is rare. The maxillary artery branches off the external carotid artery posterior to the mandibular neck, then courses anteriorly through the parotid gland and between the mandibular ramus and sphenomandibular ligament (Figure 5). 

The artery continues on deep to the lateral pterygoid muscle through the infratemporal fossa and dives into the pterygopalatine fossa 23. Structures throughout the artery’s course, such as the bony mandible and parotid gland, generally protect the artery from injury 18, 21, 23. In this patient’s case, the maxillary artery was pierced by the knife just distal to its branching point from the external carotid artery.

In contrast to CTA and MRA, POCUS is readily available at the bedside (vs. outside the patient care area), able to generate dynamic (vs. static) imaging, devoid of ionizing radiation, and cost-effective. In this patient’s case, physicians initially presumed the facial swelling to be from hematoma formation or parotid gland injury. Hematomas often appear as irregularly or regularly shaped structures, anechoic in the acute setting, but with mixed echogenicity echoes over time as the blood clots 5, 9, 24. In contrast, a pseudoaneurysm will appear pulsatile using B-mode, with the ying-yang sign using color Doppler, and showing pulsatile flow using pulsed wave Doppler 5, 9, 25, 26. The arterial wall defect can also be visualized by tracing the pulsatile flow to its origin. As opposed to a pseudoaneurysm, a simple hematoma does not typically result from ongoing high-pressure pulsatile flow leading to continuous growth. Therefore, it only warrants simple compression at most, whereas management of pseudoaneurysms requires drastically different considerations.

Management of pseudoaneurysms can be divided into non-invasive and invasive methods. Noninvasive methods include observation and trials of direct compression at the pseudoaneurysm’s neck. The goal is to eliminate flow towards the aneurysmal sac for 15-30 min to promote spontaneous thrombosis and closure of the arterial wall defect 27. The success rate across multiple studies demonstrates a 60-90% success rate, with complications including multiple attempts and cessation due to pain 27, 28, 29. Invasive methods consist of percutaneous embolization, endovascular embolization, and open surgical exploration with arterial ligation. Percutaneous embolization involves direct thrombin injection via ultrasound guidance into the pseudoaneurysm sac to promote thrombus formation 27. Endovascular embolization uses embolic agents to temporarily or permanently occlude the vessel and promote thrombus formation 30, 31, 32, 33. There is no definitive consensus for the management of pseudoaneurysms 34. In this patient’s case, the location of the pseudoaneurysm on the face was not conducive to surgical exploration. Moreover, the early success with the tight compression bandage led consulting services to opt for a trial of non-invasive management.

## Disclosures

All authors report no disclosures related to this work.

## Patient Consent

The authors obtained informed consent from the patient. 

## Supplementary Material

 Video S1 An anechoic, pulsatile, rounded, 2x2 cm structure with adjacent irregular pockets of internal echoes.

Video S2 The “ying-yang” sign – a red and blue swirling pattern that appears from pulsatile blood being ejected from the arterial wall defect into the pseudoaneurysm sac, and then redirected back towards the neck by the surrounding fibrous tissue. The gain is properly set high enough to detect the change in Doppler shift from blood flow, but not too high that would result in appearance of artifact.

Video S3 The pseudoaneurysm compressed and decreased in diameter, then once again expanded as pressure upon the distributing artery was released.
